# Coding and Classifying Knowledge Exchange on Social Media: a Comparative Analysis of the #Twitterstorians and AskHistorians Communities

**DOI:** 10.1007/s10606-020-09376-y

**Published:** 2020-06-29

**Authors:** Anatoliy Gruzd, Priya Kumar, Deena Abul-Fottouh, Caroline Haythornthwaite

**Affiliations:** 1 Ted Rogers School of Information Technology Management, Ryerson University, 350 Victoria Street, Toronto, ON M5B2K3 Canada; 2 Social Media Lab, Ted Rogers School of Management, Ryerson University, 10 Dundas Street East, Toronto, Ontario M5B2G9 Canada; 3 Faculty of Information (iSchool), University of Toronto, 140 St. George Street, Toronto, Ontario M5S3G6 Canada; 4 School of Information Studies, Syracuse University, 343 Hinds Hall, Syracuse, NY 13244-1190 USA

**Keywords:** AskHistorians, Content analysis, Knowledge exchange, Machine learning, Reddit, Social media, Twitter, #Twitterstorians

## Abstract

As social media become a staple for knowledge discovery and sharing, questions arise about how self-organizing communities manage learning outside the domain of organized, authority-led institutions. Yet examination of such communities is challenged by the quantity of posts and variety of media now used for learning. This paper addresses the challenges of identifying (1) what information, communication, and discursive practices support successful online communities, (2) whether such practices are similar on Twitter and Reddit, and (3) whether machine learning classifiers can be successfully used to analyze larger datasets of learning exchanges. This paper builds on earlier work that used manual coding of learning and exchange in Reddit ‘Ask’ communities to derive a coding schema we refer to as ‘learning in the wild’. This schema of eight categories: explanation with disagreement, agreement, or neutral presentation; socializing with negative, or positive intent; information seeking; providing resources; and comments about forum rules and norms. To compare across media, results from coding Reddit’s AskHistorians are compared to results from coding a sample of #Twitterstorians tweets (*n* = 594). High agreement between coders affirmed the applicability of the coding schema to this different medium. LIWC lexicon-based text analysis was used to build machine learning classifiers and apply these to code a larger dataset of tweets (*n* = 69,101). This research shows that the ‘learning in the wild’ coding schema holds across at least two different platforms, and is partially scalable to study larger online learning communities.

## Introduction

Conversational platforms such as social networking, microblogging, and question and answer sites (Facebook, Twitter, Reddit, Quora, Stack Overflow) provide spaces for sharing resources, engaging in ongoing exchanges, working out answers, and debating and arguing positions. Common among all these platforms is the aim of informing and being informed on topics of personal, career, and societal interest. In many cases these sites become adjuncts to career by providing open forums for questions and answers relating to the workplace, e.g., in how Stack Overflow provides a space for asking about and learning coding practices. Designing for successful online learning communities includes both a social and technical component. While the various media support online interaction, the emergent social organization and interactions show how the technologies are supporting learning.

In examining new online learning practices, we go back to some of the foundational literature on CSCW. In their first quarter century review of CSCW, Schmidt and Bannon (2013) reference an early comment by Irene Greif (1988, p. 7) that ‘[t]ransaction-oriented database systems rely on ‘coordination technologies’ for concurrency and access control and coordination’, however the ‘coordination tools are in the hands of a database administrator rather than of the end-user, and are used more to keep people from inadvertently corrupting data than for the positive goal of having a workgroup build something together’. So, too, the practice of learning has been changing from being rooted in the hands of instructors and educational administration, with the aim of coordinating access and authenticating knowledge, to the goal of learners shaping the learning environment, having a learning group building something together whether in the form of new knowledge, new learning practices, or new learning partnerships. Moreover, as learning increasingly ‘leaves the classroom’, it becomes an adjunct to daily work: open source development is complemented by open learning.

It is in this context that we direct our attention to the learning that is happening in open, online environments. In looking at learning in online settings, we find most of the research to date has focused on practices associated with formal educational settings. Much of the research on online education has focused on understanding the best methods for formal online courses to convey information, engage students, and achieve learning outcomes, aiming to initiate and improve educational and learning outcomes (e.g., Andrews and Haythornthwaite 2007; Haythornthwaite et al. 2016; Haythornthwaite and Gruzd, 2012). However, with increasing evidence of day-to-day learning online, research on social media and learning is rapidly expanding (Chugh and Ruhi 2018; Gruzd et al. 2018; Gruzd and Conroy 2020; Paulin and Gilbert 2016). As summed up by Hoadley and Kali (2019, p. 30) about learning in a networked society, ‘[t]he understanding that social scientists glean from the study of spontaneous technology-enhanced communities is a powerful force in directing our attention to learning that may occur incidentally within online communities … offering new interpretations of learner interactions and inspiring new ways to conceive of designed learning environments’.

This paper takes as its starting point a social science project examining the spontaneous learning communities of Reddit ‘Ask’ groups (also known as *subreddits*). An in-depth, multi-coder effort led to the development of a coding schema based on repeated patterns of exchange relating to learning and community in the ‘Ask’ subreddits: AskHistorians, Ask_Politics, AskScience, and AskAcademia. This ‘learning in the wild’ coding schema follows the efforts of others in coding learning discourse (e.g., Gunawardena et al. 1997; Ferguson et al. 2013), and was finalized over three rounds of evaluation. The coding schema is described in more detail below, but in brief the eight derived categories are: (1–3) Explanation with Disagreement, Agreement, or Neutral Presentation; (4–5) Socializing with Negative, or Positive Intent; (6) Information Seeking; (7) Providing Resources; and (8) forum Rules and Norms (see Kumar and Gruzd 2019; Kumar et al. 2018; Haythornthwaite et al. 2018).

While the coding schema proved useful for evaluating exchanges in Reddit, the questions remained: Would the coding schema hold for another medium, such as Twitter? And, if the coding schema held, how different are interactions in the two different media? Further, if the coding schema held, can the human coding process be scaled up into an automated process to evaluate greater sample sizes? These questions lead to our research questions, and the outline of our study.

### Applying the coding schema to Twitter

- RQ1: Does the ‘learning in the wild’ coding schema developed from Reddit ‘Ask’ subreddits hold for another social medium?

To address whether the coding schema holds for another medium, the schema was applied to the Twitter learning community associated with the hashtag #Twitterstorians. This hashtag community brings together self-described ‘history buffs’ into a community designed to sustain information sharing and communication about history. This community was also selected because the coding schema was created in part on Reddit’s AskHistorians site, another history discussion site, thereby facilitating comparison across media.

### Comparing across media

- RQ2: How do the types of information, communication, and discursive practices of a Twitter-based discussion and learning community compare with a similar Reddit-based community?

Finding that the schema held, results for #Twitterstorians were then compared to those of AskHistorians. Data for Twitter was newly gathered for this study, while Reddit data and analysis were drawn from the work in the previous study by Haythornthwaite et al. (2018).

### Scaling up with machine learning techniques

While manual coding can be effective, the quantity of data generated in social media calls for an automated process. Thus, the third phase of this research was to develop, apply and evaluate machine learning techniques for coding #Twitterstorians data.

- RQ3: What is the effectiveness of machine learning techniques in detecting different codes of the ‘learning in the wild’ schema on Twitter?
- RQ4: What are the main linguistic features that help predict different codes of the ‘learning in the wild’ schema on Twitter?

Results discussed below confirm that the proposed coding schema can be partially scaled to analyze large size datasets with enough accuracy to be confident in the automated coding process. This was achieved for a subset of four codes included in the machine learning-based analysis for which sufficient cases existed (described below).

The current study makes a number of contributions to the CSCW literature (Wobbrock and Kientz 2016): *theoretical* by proposing the ‘learning in the wild’ coding schema to study different types of knowledge construction and discursive practices being supported on social media; *empirical* by applying the proposed coding schema to analyze the hashtag-based online learning community associated with the hashtag #Twitterstorians and comparing results to data from Reddit; and *methodological* by developing, applying and evaluating how well machine learning techniques scale the analysis to large size datasets. The following sections provide background on theories and studies on interaction in support of learning online (Section 2), outline the ‘learning in the wild’ coding schema (Section 3), and our method (Section 4). Section 5 presents results relating to the #Twitterstorians coding, comparison with AskHistorians, and machine learning evaluation.

## Learning online

A number of theoretical perspectives underpin research on learning in online settings. Research on *collaborative learning* has been particularly focused on the way interaction facilitates peer observation, evaluation, and learning, building into the extensive research in computer-supported collaborative learning (CSCL; Bruffee 1993; Koschmann 1996; Koschman et al. 2002; Stahlet al. 2006; Buckingham Shum and Ferguson 2012; Paulin and Haythornthwaite 2016).

The theory of *social learning* underpins research that examines how interaction supports learning outside formal settings. Social learning emphasizes the learning that happens through observation of behaviors, and how the learner chooses to imitate (or not) the behavior depending on observation of reactions to that behavior (Bandura 1977; Buckingham Shum and Ferguson 2012). Apprenticeship learning is similar, but highlights the role of experts, with novices learning by observing and engaging in Communities of Practice (CoPs; Lave and Wenger 1991). Interaction allows learners to bridge the *Zone of Proximal Development* with the help of teachers, experts, or ‘more knowledgeable others’ (from the ideas of Lev Vygotsky; Gunarwardena et al. 2009; Goos 2002; Paulin and Gilbert 2016). Learning communities show a variety of interactive learning patterns, such as the kinds of talk identified by Mercer (2004) as promoting learning in a classroom setting: Disputational (disagreement and individualised decision making), Cumulative (building positively but uncritically on what the others say), and Exploratory (engaging critically but constructively with others’ ideas). Observing and practicing *argumentation* plays a large role in interaction in peer-based learning, determining what is persuasive, what is considered a good or useful presentation of opinion or facts, and how to apply that effectively (Andrews 2009; Wise et al. 2014; Khazaei et al. 2017; Gilbert 2018; Sengupta and Haythornthwaite 2019).

### Learning on and through Twitter

Learning through Twitter can happen through self-directed learning, e.g., by seeking out a Twitter community for learning. Such was the case for the #hcsmca (Health Care Social Media Canada) community, where community members gathered for tweetchats (synchronous Twitter engagement) to learn and exchange views on applying social media to health care in Canada (Gilbert 2016). In other instances, Twitter becomes an adjunct to a formal learning environment. Such is the case for the kinds of Massive Open Online Courses (MOOCs) given by George Siemens, where individuals were encouraged to use social media to make connections in support of their own connectivist learning (Siemens 2005). Similarly, Reed (2013) describes Twitter as supporting the 3C’s of ‘community, communication, and casual (informal) learning’, examining how individuals can use the platform to develop personal learning environments.

From the perspective of instructors – and thus potentially of those who provide instruction and explanation in open forums – Twitter presents a rich and open online environment to enhance teaching pedagogies and individual learning objectives both inside and outside formal classroom settings. For example, when instructors participate on Twitter they are not focused solely on formal instruction. Rather, they tend to use the platform to share resources with their professional networks, share information about classroom affairs, request help and assistance from others, engage in social commentary and conversations, connect with others outside of their networks, and manage their own personal teaching environments (Veletsianos 2012; Chen and Bryer 2012; Lewis and Rush 2013; Gruzd et al. 2018).

### Coding schemas for learning online

To delve into the discursive norms and practices of online hashtag communities, several studies have developed coding schemas for content analysis. Schemas have been applied to online discourse to examine knowledge construction processes by students, emergent roles found in classes, processes of argumentation, and media use differences. Perhaps best known is the Interaction Analysis Model by Gunawardena et al. (1997). The model proposes that social construction of knowledge in online environments happens through five main phases: sharing or comparing of information; discovery and exploration of dissonance or inconsistency among ideas, concepts or statements; negotiation of meaning; testing and modification of proposed synthesis; and agreement statements or applications of newly constructed meaning.

Others provide further insight into knowledge exchange and learning practices online. Examining argumentation, Pena-Shaff and Nicholls (2004) found online participant-learners used both positive (agreement) and negative (disagreement) communication in meaning-making and knowledge construction. Baker et al. (2007), examining phases of deliberation and argumentation, were able to show how conversational debates can help broaden and deepen knowledge construction. Weinberger and Fischer (2006), applying a multi-dimensional coding framework to discussion boards, found that argumentative dimensions of collaborative learning fed into social modes of knowledge co-construction, such as conflict-orientated consensus building. And, de Laat and Lally (2003), using two complementary coding schemas (those of Anderson et al. 2001; Veldhuis-Diermanse 2002), found differences across learning communities, but similar roles in participation patterns, such as a conversational facilitator role.

Lastly, we note work by Ferguson et al. (2013) which applied Mercer’s ideas of exploratory dialogue to analysis of online class discussion, deriving seven categories of dialogue that included critique, evaluation, explanation, explicit reasoning, justification, extension of others contributions, and discussion of resources. As described below, the coding schema used in this research built on past work such as those noted here, and particularly on Ferguson et al.’s work to identify a schema suitable for machine coding.

### Adding community to the coding schema

While much of the coding efforts above focuses on subject learning, Twitter-based communities share with other social media platforms and virtual communities the lack of a formal educational structure and the need to self-organize the rules for behavior. Thus, to understand the full range of learning that is going on – learning that includes defining and coming to common practices about forum purpose and practice – requires equal attention to learning and engagement with the community (Wenger 1998; Preece 2000; Gruzd and Haythornthwaite 2013; Gruzd et al. 2016).

Previous work addressing online forums often comes back to the need to organize collective action and expression in a way that allows work to get done – whether the project is commercial or educational, and whether the work to be done is to launch a product, create or deliver knowledge, or build a community (e.g., Nardi et al. 2002; Orlikowski 2002; Renninger and Shumar 2002; Hine 2006).

This background emphasizes how use of any social medium for learning engages with the need to create and sustain communal, collaborative processes for crowd-sourced peer production, with norms for communication developing through shared, cooperative interaction.

## The ‘learning in the wild’ coding schema

The ‘learning in the wild’ coding schema builds on the earlier work on both learning and community interaction, including research on argumentation, group processes, online community definition and support, online learning, and peer production communities. The schema was developed by a team of six researchers (two faculty members, two postdoctoral fellows, and two doctoral students) using data from four Ask subreddits: AskHistorians, Ask_Politics, AskScience, and AskAcademia (Kumar et al. 2018; Haythornthwaite et al. 2018). This coding schema expanded on the work of Mercer (2004) on exploratory dialogue, and its application to formal online learners by Ferguson et al. (2013). The coding effort followed the lead of these studies in considering exploratory dialogue to be an essential feature of collaborative learning and knowledge construction. While earlier rounds followed more closely the Ferguson et al.’s coding schema for learning and argumentation, the lack of fit to the open, online discourse led to a more open coding process in the final round.

The eight-item coding schema that emerged from this empirical work captures aspects of deliberation (explanations with positive, negative or neutral expression), socializing (both positive and negative), engagement in learning (seeking information, providing resources), and instruction in the rules and norms of the forum (Table 1). A strength of the final coding schema is that its open derivation focused on what constituted exchanges among forum members, rather than trying to extract the ‘learning’ components from the whole. This gave a better view of learning embedded in the communicative exchanges within the community relative to the particular medium, forum, and culture.

**Table 1:** ‘Learning in the Wild’ Coding Schema.

Code	Definition	Linguistic Dialogue Example
**Explanation with Disagreement**	Expresses a NEGATIVE take on the content of the previous posts by adding new ideas or facts to discussion thread	‘But’, ‘I disagree’, ‘not sure’, ‘not exactly’ with explanation/ judgement/ reasoning/ etc.
**Explanation with Agreement**	Expresses a POSITIVE take on the content of the previous posts by adding new ideas or facts to discussion thread	‘Indeed’, ‘also’, ‘I agree’, with explanation/ judgement/ reasoning/ etc.
**Explanation with Neutral Presentation**	Expresses a NEUTRAL explanation/ judgement/reasoning/etc. with neither negative nor positive reference to the content of the previous posts, nor necessarily any reference to previous posts	‘I can understand’, ‘interesting’, ‘depends on…’ or statement responses
**Socializing with Negative Intent**	Socializing that expresses negative affect through tone, words, insults, expletives intended as abusive	‘No’, ‘you’re an idiot’, ‘this has been explained multiple times’
**Socializing with Positive Intent**	Socializing that expresses positive affect tone, words, praise, humour, irony intended in a positive way	‘Thanks’, ‘great feedback’, ‘you’re correct’
**Information Seeking**	Postings asking questions or soliciting opinions, resources, etc. This does not include questions answered rhetorically within the post, e.g., if a question is asked and answered	‘Does anyone know?’, ‘Can anyone explain?’
**Providing Resources**	Postings that include direct reference to a URL, book, article, etc.; postings that call upon a well-known theory or the name of a well-known figure	Link to resource (book, URL, article, audio/video file). Referencing theory/theorists, scholar or public work (Einstein, Newton, Freud)
**Rules and Norms**	Postings on topics such as what is the appropriate for a particular discussion, what language is appropriate to use, how to back up claims by using resources, using hashtags, etc.	‘See/don’t forget link’, ‘this post doesn’t belong here’, acknowledging OP/HT Twitter users, hashtags and bots

Coding with the final schema applied up to three codes per post; a code was accepted only where two coders agreed. Among the four subreddits, Krippendorff’s alpha in the testing phase and by independent coders were higher for the more question and answer oriented subreddits AskScience (independent coders: 0.69, and 78% agreement), and AskHistorians (independent coders: 0.76, and 79% agreement); and lower for discussion-type subreddits: Ask_Politics (independent coders: 0.60 and 72% agreement), and AskAcademia (independent coders: 0.64 and 77% agreement). Given the exploratory nature of the study and the application of multiple codes, the alpha levels and agreement among independent coders were considered reliable enough to draw out and develop cautionary conclusions (alpha levels between 0.67 and 0.80 are recommended with single codes; Hayes and Krippendorff 2007; Krippendorff 2004).

Overall, the coding schema fit was best for AskHistorians, and thus represents the most reliable basis for the objectives of the current research of testing and validating this coding schema for Twitter and deriving an automated process for coding.

## Data collection and analysis

Data for application of the coding schema to Twitter consisted of all publicly available #Twitterstorians tweets (original posts and replies) over a 30-day period from June 20 to July 20, 2017, captured using Netlytic (https://netlytic.org/), an online program for social media text and network analysis. A total of 17,391 Twitter messages were collected by querying Twitter’s Search API and retrieving up to 1000 #Twitterstorians tweets every 15 min; after removing duplicates and retweets the dataset was comprised of 6349 tweets. To create a sample set for coding, 10% of these tweets were used by selecting every 10th tweet when sorted chronologically, for a final sample of 633 tweets that were then manually coded using the ‘learning in the wild’ coding schema.

Next, we compared the information, communication, and discursive practices observed in the Twitter-based discussion, as demonstrated in the presence and prevalence of codes from the schema, with that of the similar Reddit-based community AskHistorians, as collected and analyzed in earlier work. The earlier Reddit sample was derived from all submissions and comments posted to AskHistorians group (subreddit) in 2016. In total, there were 41,214 submissions (threads) containing 142,279 comments. After excluding comments deleted either by the authors or the moderators (because the data was collected retroactively), the remaining number of comments were 122,670. We then took the first 1% of comments *n* = 1227 as our Reddit sample for evaluation. As in case of the Twitter sample, the Reddit sample was manually coded by three independent coders using the ‘learning in the wild’ coding schema.

For the machine learning part, we collected a larger sample of #Twitterstorians public tweets consisting of 69,101 original posts and replies from 2018 (excluding RTs). In order to evaluate a much larger dataset, multiple machine learning classifiers were built, and applied to this dataset. The first part of this process is to extract features from text to be used during the training step. To avoid the possibility of making our classifier too domain specific, we relied on the Linguistic Inquiry and Word Count (LIWC) software tool to move from using actual words or n-grams from text to a higher level of abstraction. LIWC does this by detecting and grouping words from text into 94 broad linguistic features that represent various emotional, cognitive, and structural components in written text (Tausczik and Pennebaker 2010). This abstraction process also allowed us to reduce the number of features used for training our classifiers (Abaho et al. 2018). LIWC has been successfully applied to create models to classify tweets in prior studies in areas such as mental health (Zhao et al. 2019), food consumption (Abbar et al. 2015), and political discussions (Sylwester and Purver 2015). A Random Forest model was used for feature selection. Nodes with the greatest decrease in impurity happen at the start of the trees, while nodes with least decrease in impurity happen at the end. Pruning trees below a particular node creates a subset of important features. We applied pruning to only keep important features that lead to a larger mean decrease in Gini coefficient over the various trees within the Random Forest. After going through feature selection using Random Forest, important features were kept and used to build the classifiers. Models are trained and tested based on a 70:30 random distribution of training and test datasets. Training and test datasets have the same distribution of classes as the original dataset. Models are then validated using five repeats of 10-fold cross-validation. Once trained and validated, the resulting classifiers were applied to categorize all 69,101 tweets.

**Figure 1:**
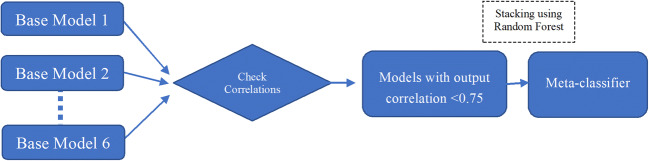
Process of Building Classifiers Using Ensemble Stacking Technique.

To ensure building robust classifiers, we followed two steps. First, we applied a collection of six base sub-models: Random Forest (RF), Support Vector Machine (SVM), Gradient Boosting Machine (GBM), Naïve-Bayes, Logistic Regression (LR), and Tree Bagging (TB). These models are commonly used in relevant literature on classification of Twitter text (Guzman et al. 2017; Stanik et al. 2019; Williams and Mahmoud 2017). We eliminated highly correlated base models (correlation values higher than 0.75). Second, after eliminating highly correlated models, a meta-classifier using stacking of the remaining base models was built using Random Forest on the predictions of the base models (see Figure 1). We built supervised machine learning classifiers for tweets where there were enough instances for training and applying the codes. This resulted in a set of four codes – Explanation with Neutral Presentation; Socializing with Positive Intent; Information Seeking; Providing Resources – and excluded all others.

To assess the performance of the resulting models, and mindful that accuracy alone might not be enough to determine the performance of imbalanced models (Flach and Kull 2015), we used four different measures: accuracy, precision, recall, and F1 score. *Accuracy* refers to the overall effectiveness of a classifier. It is the number of instances (in our cases, tweets) correctly classified as not a member of (positive) or a member of the class (negative), divided by the number of all instances labeled by the system (Sokolova and Lapalme 2009). *Precision* is the proportion of true positives among the positive attributions made by the classifier. *Recall* is the proportion of true positives among all relevant positives (regardless whether they were identified by the classifier or not). The *F1 score* combines precision and recall into a single performance measure, commonly known as the harmonic mean of precision and recall (Sokolova and Lapalme 2009). This score is widely used in machine learning studies as it balances quality versus quantity of the true positive selection (Nayak and Natarajan 2016).

## Results

- RQ1: Does the ‘learning in the wild’ coding schema developed from Reddit ‘Ask’ subreddits hold for another social medium?

The manual coding was done by two independent coders, one post-doctoral fellow and one graduate research assistant, each of whom completed a schema tutorial training module prior to commencing the coding process. Of the initial sample, 94% (594 of 633) of the tweets were successfully coded using the ‘learning in the wild’ schema (i.e., two coders agreed on the code).

Results from the manual coding part showed an acceptable level of agreement between the two coders: Krippendorff’s alpha of 0.65 and intercoder agreement of 73%. The resulting alpha of 0.65 is close to a recommended threshold of 0.67 to be considered reliable enough for exploratory studies like ours (Bolognesi et al. 2017; Hayes and Krippendorff 2007; Krippendorff 2004).

These results are in line with previous applications of the ‘learning in the wild’ coding schema; and in line with Ferguson et al.’s (2013) binary coding of online conference dialogue (exploratory and non-exploratory) which recorded an intercoder agreement score of 0.597 (indicating ‘moderate agreement’ sufficient enough to train an automated classifier). At the same time, because our coding schema is not binary, and allows for a maximum of three codes per post, a lower agreement among coders would be expected. For example, while both coders labelled 223 posts as Providing Resources, in 25 cases only one of the coders also labeled tweets as Socializing with Positive Intent. This happened when a user shared a resource with an explicit endorsement and/or expression of excitement about its content. Here is a sample tweet: ‘An incredibly important short piece on how #twitterstorians can be impactful. https://t.co/zxNSYVtm5D via @chronicle’. Another example of disagreement is when 23 Information Seeking posts (out of 100) were also coded as Providing Resources by one coder, but not another. This happened when a post was framed as a question but also contained a URL presumably linking to a resource with an answer, such as ‘What kind of people joined military orders like the Templars and the Teutonic Knights? https://t.co/CYJ2C2zcYc #twitterstorians’.

For 6% (39 of 633) of the sample, the coders agreed that the tweets did not fit into any of the ‘learning in the wild’ codes. A review of the codes suggests they constitute another type of information exchange on Twitter, one that might be referred to as ‘networking about events’. All but a few of these tweets were short announcements about events or opportunities to network with other #Twitterstorians. For example:

- ‘Check out the New Urban History Dissertation Group Monthly workshop @NewberryLibrary https://t.co/U63NXQrWUd #twitterstorians’
- ‘This looks like a great opportunity for scholars working on early American women and religion. #twitterstorians #amrel’
- ‘Go behind the scenes of the new @MusCanHistoire’s Hockey Exhibit! https://t.co/BG58s5nURQ #twitterstorians #cdnhist #cdnhistory #hockey’

While this is an important new category that can be introduced in future research, it was not included in the comparisons or machine learning below because the code has not been fully defined or agreed between coders.

### Comparing #Twitterstorians and Reddit AskHistorians

- RQ2: How do the types of information, communication, and discursive practices of a Twitter-based discussion and learning community compare with a similar Reddit-based community?

To examine this question, the information, communication, and discursive practices as demonstrated in the presence and prevalence of codes from the applied ‘learning in the wild’ schema, were compared across the two learning communities. Table 2 shows the distributions of codes for #Twitterstorians and Reddit AskHistorians. Percentages add up to over 100% in each case because coders could apply up to three codes per tweet. Tweets were manually classified under a particular schema code only if the two coders agreed, and percentages are rounded to the nearest 1%. (We note again the 39 tweets that appear to provide clues to another category of ‘networking events’ are not included in this discussion; we leave verification and definition of this potential new code to future research.)

**Table 2:** Manual Coding Results: #Twitterstorians vs Reddit’s AskHistorians.

	#Twitterstorians (n = 594)	Reddit AskHistorians (*n* = 1227)
Explanation with Disagreement	3 (1%)	71 (6%)
Explanation with Agreement	4 (1%)	45 (4%)
Explanation with Neutral Presentation	73 (12%)	592 (48%)
Socializing with Negative Intent	1 (0%)	4 (0%)
Socializing with Positive Intent	99 (17%)	204 (17%)
Information Seeking	100 (17%)	274 (22%)
Providing Resources	223 (38%)	260 (21%)
Rules and Norms	22 (4%)	66 (5%)
Krippendorff’s Alpha	0.65 (73%)	0.76 (79%)

*Messages were classified under a particular code only if the two coders agreed. Percentages add up to over 100% because coders could assign up to three codes per message. Percentages are rounded to the nearest 1%

The distribution results show Twitter posts are concentrated on Explanation with Neutral Presentation (12%), Socializing with Positive Intent (17%), Information Seeking (17%), and then a large proportion relating to Providing Resources (38%). Very low instances of negative affect were found, i.e., Explanation with Disagreement, or Socializing with Negative Intent; and Explanation with Agreement was also low.

Comparing interaction patterns of #Twitterstorians and AskHistorians shows some key similarities and differences which may be the result of the affordances of these two forums. Overall the platforms share similarities in community dialogue that is transactional and functional, both showing high proportions of Information Seeking (17% for Twitter; 22% for Reddit) and Providing Resources (38% and 21%), in keeping with the Q&A nature of both platforms. Both sites show strong positive, and low negative socializing, which suggests that both online platforms are supporting socially positive learner conversations even though Reddit is a predominantly anonymous environment (for more qualitative analysis of the AskHistorians culture within the Reddit setting, see Gilbert 2018). The positive socializing also suggests that both platforms support active historian communities, where members connect based on similar interests or goals, and strive to learn from one another through social media. This aspect of sharing is also evident in the communication of networking events in the 39 uncoded tweets.

The codes of Explanation with Neutral Presentation, and Providing Resources differ most in proportion between Twitter and Reddit, with much more explanation on Reddit (12% on Twitter compared to 48% on Reddit), and less provision of resources, although this remains a major activity on Reddit (21%). These results appear to be in line with the affordances and practice of tweeting which favors short over long postings by design (indeed to post a long comment in Twitter requires multiple tweets), where learners use the ‘shorthand’ of a reference over longer deliberation or explanation.

Differences in the platforms extend to both the interface design and social behaviors. The text limit differences between Twitter (280 characters) and Reddit (15,000 characters) create different expectations of question and answer behavior, which then shape the opportunities and motivations for participation. Our coding results show a higher proportion of posts on Reddit with all three types of explanation (with disagreement, agreement, and neutral presentation). While the text limit offers one potential explanation, compared to Twitter, Reddit may be more inviting for participants looking to ask in-depth questions, and/or thoroughly explain their thoughts about a particular issue with fellow community members (Haythornthwaite et al. 2018). Further, the anonymity of the Reddit platform promotes blind ‘peer review’ through its upvote/downvote system. Rewarding Redditors based on the quality of their posts (known as ‘karma’) might entice members to put forth well thought-out commentary. Finally, differing from Twitter, each subreddit community is maintained by a group of moderators that administers a unique set of rules and norms that function as a code of conduct (known as ‘Reddiquette’) for community members to follow (Del Valle et al. 2020; Gilbert 2018; Loudon 2014). All three mechanisms – longer postings, up/down voting and karma points, and moderator interceptions – support informal learning about rules and norms, and thus about how to participate in the subreddit. Moreover, as Gilbert (2018) reported following interviews with AskHistorian participants, one of the observations made by novices is that they learn about historiography, i.e., how history is practiced, allowing participants to also come to know about the practices of the epistemic community; the explanatory practices fostered on Reddit appear to provide a more in-depth learning environment, where elements of historiography, rather than just history facts and references, can be observed and learned informally.

By contrast, the Twitter platform promotes much shorter (under 280 characters) and more public forms of conversational dialogue (Kwon et al., 2014). Hashtags (such as #Twitterstorians) bind and connect texts and users, and help maintain a sense of community between an otherwise dispersed network of individuals and tweets. For these reasons, deliberative processes and exploratory dialogue on Twitter can be expected to be more to the point, with resources and information that is easy to find, follow, digest, and share (i.e., Information Seeking, and Providing Resources). The hashtag is an essential affordance of Twitter and for #Twitterstorians. As a technological feature, and as a social feature adopted to index tweets, hashtags perform an essential function for finding topics and people and sifting through resources in a crowd of contributions and learners, bypassing the noise of Twitter.

We also observed that in Providing Resources through #Twitterstorians, people often linked to conversations happening in other platforms such as Reddit. Twitter then becomes one element of an individual’s personal learning environment, managed in a connectivist fashion (Siemens 2005) by linking to other people, resources, and platforms. Cross-connections such as these between different people and platforms provide Twitter users with access to a wider set of contacts, contexts, and resources, nurturing higher levels of bridging social capital and opportunities to connect with loose social network ties (Shane-Simpson et al. 2018).

### Scaling the ‘learning in the wild’ schema

- RQ3: What is the effectiveness of machine learning techniques in detecting different codes of the ‘learning in the wild’ schema on Twitter?

**Figure 2:**
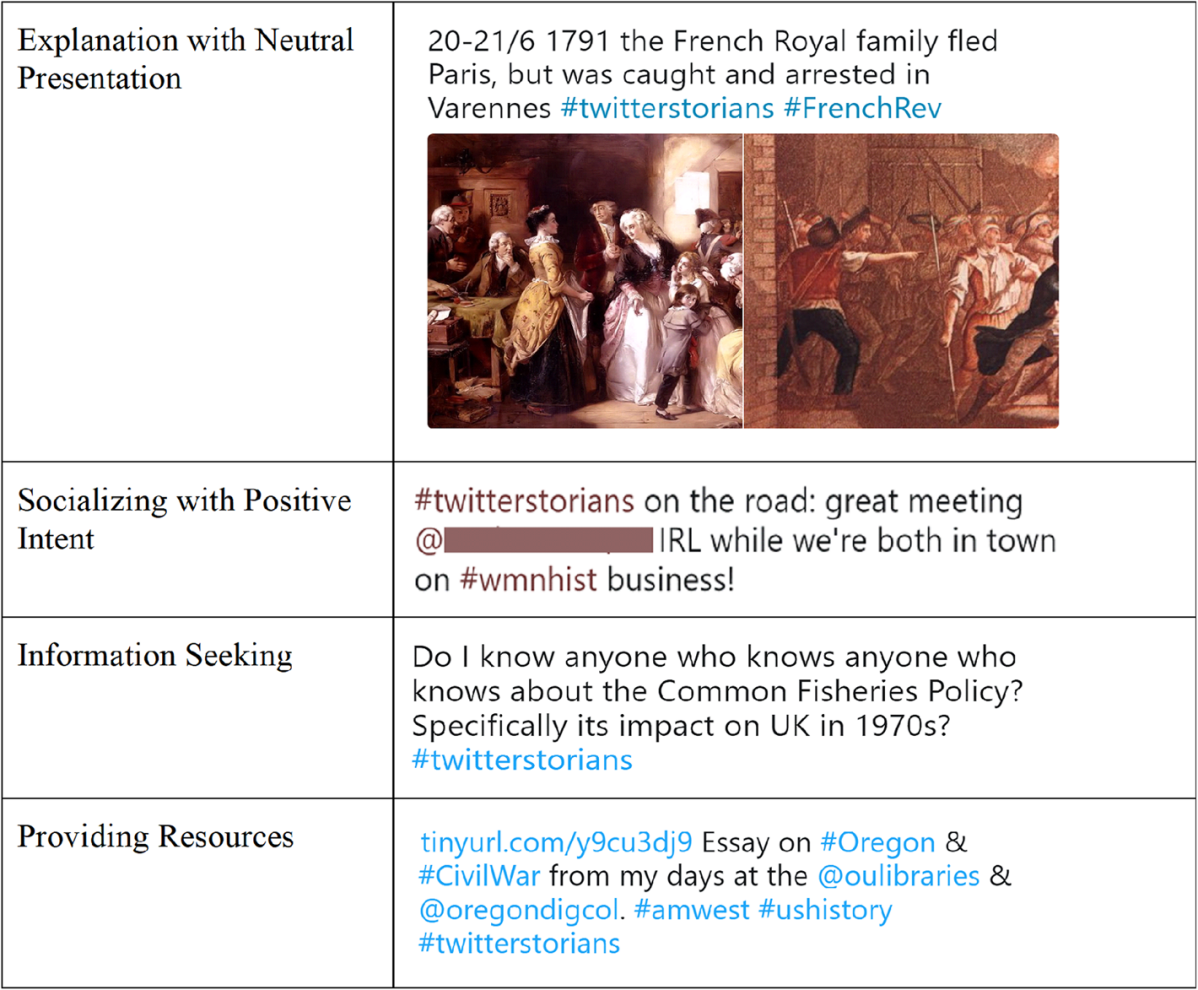
Examples of Tweets for Frequently Found Categories.

The overall results of our automated coding confirmed that the trained classifiers performed well across all four measures: accuracy, precision, recall and F1 score. For each code, we used the classifier (or an ensemble of classifiers) that showed the best performance when predicting the occurrence of this code in the 2017 Twitter dataset. Only codes that appeared in more than 10% of the tweet dataset were used (a commonly used threshold for training of classifiers). These are: Explanation with Neutral Presentation; Socializing with Positive Intent; Information Seeking; Providing Resources (see Figure 2).

Appendix 1 provides the mean accuracy over the ten folds of the ensemble model and the base models after removing highly correlated models. Results show that the ensemble model performed better than all the base models for all the classifiers.

Table 3 provides a breakdown of the performance measures of the ensemble models. The classifiers performed particularly well compared to another similar study (Birnbaum et al. 2017), which also applied LIWC categories to build classifiers using Twitter data. In their study detecting markers of schizophrenia on Twitter, the classifier had the accuracy, precision and recall values of 0.59, 0.27, 0.77 (based on unseen data). While the application area of the Birnbaum et al.’s study is different from ours, their findings generally suggest an acceptable level of our results considering the exploratory nature of this work. However, we note the F1 score for the ‘Socializing with Positive Intent’ code is particularly low, which we hope to address with more training data in future work.

**Table 3:** Performance Measures of Machine Learning Classifiers.

Code	Classifier	Mean Accuracy over Ten Folds	Accuracy Standard Deviation over Ten Folds	Precision	Recall	F1 Score (Harmonic Mean)
Explanation with Neutral Presentation	Stacking of RF, SVM, Tree Bagging, LR, and GBM	0.9164	0.0109	0.9000	0.4091	0.5625
Socializing with Positive Intent	Stacking of RF, SVM, Tree Bagging, LR, Naïve Bayes, and GBM	0.8836	0.0178	0.4615	0.207	0.286
Information Seeking	Stacking of RF, SVM, Tree Bag, LR, Naïve Bayes, and GBM	0.9750	0.0106	0.8500	0.5667	0.6800
Providing Resources	Stacking of RF, SVM, Tree Bagging, LR, and Naïve Bayes	0.8728	0.0192	0.7308	0.5588	0.6333

When we applied the trained classifiers to analyze the large dataset of tweets from 2018, we found that there is a consistency in the resulting distributions of codes when compared to the manually coded dataset from 2017, with differences within 5% (see Table 4). This relative similarity in the distribution of the four codes between the two datasets suggests that the types of tweets that the community members posted in 2017 and 2018 remained relatively similar.

**Table 4:** Distribution of ‘Learning in the Wild’ Codes in 2017 versus 2018 Datasets

	Manual coding	Machine learning
	% of tweets in the 2017 dataset (n = 594)	% of tweets in the 2018 dataset (n = 69,101)
Explanation with Neutral Presentation	12%	7%
Socializing with Positive Intent	17%	15%
Information Seeking	17%	17%
Providing Resources	38%	33%

To understand further why tweets were classified under different codes during the machine learning part, we reviewed the dominant predictors. As discussed above, the predictors came from the list of 94 emotional, cognitive, and structural features available in LIWC. In our experiments, the features that have consistently shown to be useful when classifying tweets came from the following top-level categories: Linguistic Processes, Other Grammar, Psychological Processes, Time Orientations, Personal Concerns, and Punctuation (see Appendix 2).

**Figure 3:**
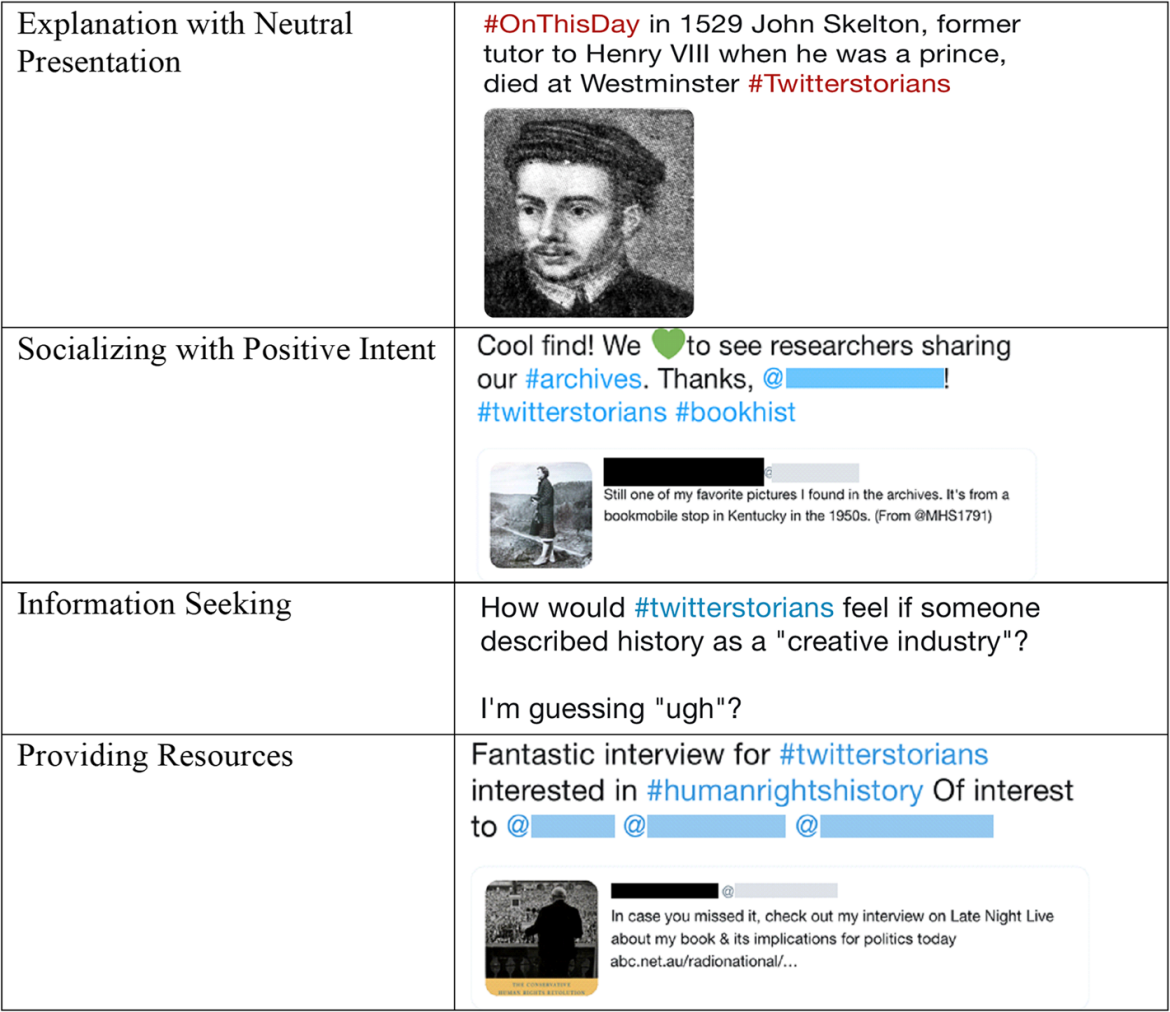
Sample Tweets as Categorized.

The following highlights some of the most representative linguistic features for each code. For Explanation with Neutral Presentation, the presence of *Past focus* and *Death* features among the strongest predictors accords with the #Twitterstorians emphasis on past events and the lives of historic figures. Figure 3 shows a sample tweet in this category that contains words such as ‘former’, ‘was’, and ‘died’.

For Socializing with Positive Intent, the presence of predictors such as *First person singular pronoun ‘I’, Positive emotion, Present focus, and Exclamation marks* is reassuring because words from these features are often associated with informal and welcoming posts. Figure 3 shows a sample tweet with words such as ‘cool’ and ‘thanks’.

For Information Seeking, interrogatives (e.g., ‘how’ or ‘why’), question marks, and some level of uncertainly (*Tentative* feature) in a tweet were among the strongest predictors of this code. The presence of these linguistic features is indicative of a user soliciting information from other community members. The result also suggests that community members frequently use informal language and netspeak when asking questions. Figure 3 shows a sample tweet for this code.

As the types of resources shared by community members varied significantly, so do the features that predict Providing Resources. Without listing all applicable features here (see Appendix 2 for the full list), the presence of features such as *Analytic*, *Clout*, and *Authentic* speaks to knowledgeable responses to inquiries from others. Also, as for ‘Information Seeking’, we observe the importance of such features as *Informal language*, *Netspeak*, and *Social processes*; all signs of the #Twitterstorians community being a friendly, informal group where people openly share resources with others. See Figure 3 for a sample tweet categorized with this code.

## Conclusions

In this work, we analyzed the hashtag-based online learning community associated with the hashtag #Twitterstorians to understand and assess the different types of knowledge construction and discursive practices being supported on Twitter. We applied the ‘learning in the wild’ coding schema, first developed to examine learning and interaction patterns in Reddit, to a sample of public tweets posted with the hashtag #Twitterstorians. In doing so, we evaluated whether this coding schema could reliably capture the discourse, talk, and social cues that promote exploratory dialogue in social media.

In the manual coding stage, two independent coders applied the schema to #Twitterstorians tweets, achieving an intercoder agreement of 73%. Our results showed that Twitter affords new networked opportunities for participant-learners outside formal educational settings. More specifically, we found that the #Twitterstorians community sustains itself through socially positive information and resource exchanges. Differences between the results for #Twitterstorians and past results for Reddit’s AskHistorians (notably the much lower proportion of text coded as ‘Explanation’) suggest how different interface affordances may promote different forms of social and learning interaction. For example, Twitter’s character limit encourages community exchange that is short and ‘to the point’, favoring providing references over explanation. In terms of learning, the two sites offer a different, but complementary approach to information seeking and provision.

As a learning community, #Twitterstorians reflects several perspectives from the literature on learning. Exchanges provide the opportunity to connect to other people and other resources, supporting ideas of connectivist learning (Siemens 2005), and Twitter as a component in an individual’s personal learning environment (Reed 2013). Collaborative learning is demonstrated in providing explanations, but the short-format tweets appear to favor a more pass-through approach of pointing individuals to other sources. As such, one might say Twitter acts more as an information kiosk than a classroom, directing others to resources far more often than teaching others. A more in-depth look at the explanation texts may illuminate further whether these are reference interview or teaching moments. Finally, #Twitterstorians shows itself as a socially positive community, providing the ‘safe space’ for learning described as needed for collaborative learning.

To move forward with analyzing learning and community exchanges online requires the ability to analyze much larger datasets than possible with manual coding. To this end, this research used a supervised machine learning approach to detect the ‘learning in the wild’ codes. Specifically, we trained machine learning classifiers to detect the four codes with enough instances from the manually coded dataset (>10%). Our classifiers performed well (see Table 3), indicating that the four codes from the ‘learning in the wild’ coding schema, for which there were sufficient examples to test, are scalable to study larger online learning communities, namely: Explanation with Neutral Presentation, Socializing with Positive Intent, Information Seeking, and Providing Resources. For these four codes, our results also demonstrated consistency over time in the types of information, communication and discursive practices used in the #Twitterstorians learning community in 2017 (based on a sample of 594 original tweets and replies) and in 2018 (based on all 69,101 tweets and replies). However, to test the scalability of the complete ‘learning in the wild’ schema, a substantially larger sample of manually coded social media posts are needed to train and evaluate machine learning classifiers for low frequency events such as Explanation with Disagreement, Explanation with Agreement, Socializing with Negative Intent, Rules and Norms.

We intend to expand this research, first by applying and further validating the schema across other social media platforms (such as Facebook and YouTube), and exploring further possible additions to the schema such as the inclusion of an additional code capturing instances of Networking about Events. We then intend to invite instructors who use social media like Twitter for teaching to test the schema in order to more precisely evaluate the learning, socializing, and collaborative practices that increasingly play a role in both formal and informal learning environments.

Although our experiments showed that using LIWC to extract features for training can produce reliable classification results, future work will apply more advanced feature extraction techniques such as Latent Dirichlet Allocation for topic modeling that have been shown to produce even more accurate results (Resnik et al. 2015). Also, many of our trained classifiers appeared to be specific to the historical domain. For example, some of the codes relied on words from the *Past focus* and *Death* LIWC features, which may or may not be applicable when classifying tweets from other domains. We plan to further complement this research by analyzing tweets from Twitter communities that discuss different topics. In this regard, techniques such as domain adaptation, transfer learning, active and online learning could help models to adapt to new domains and address issues associated with the lack of training data (Johnson et al. 2020; Kaufhold et al. 2020; Stowe et al. 2018).

Finally, while this work focused on the analysis of individual posts, we see an opportunity to expand the proposed schema to account for how posts are sequenced and who is replying to whom. Beyond solely examining distributions of different message types, adding an additional layer of analysis by looking at sequences of interaction events may highlight nuances in online learning practices. Such analysis would allow us to answer additional research questions about common patterns of interaction in online learning communities, and facilitate prediction of post types based on a chain of previously posted message types. Answers to questions like these could then feed into the system design process, for example, by developing affordances to inform moderators of online groups when a conversation might be going off track and there is a need to intervene, acting as a form of an early alert system to maintain civil and constructive online discussions.

## Appendix 1

**Table 5:** Comparing Performance across Classifiers

Classifier Model	Mean Accuracy over Ten Folds	Accuracy Standard Deviation over Ten Folds
Code: Explanation with Neutral Presentation		
RF	0.8983	0.0226
SVM	0.9065	0.0240
Tree Bagging	0.8954	0.0266
LR	0.8974	0.0308
GBM	0.9007	0.0282
Stacking of RF, SVM, Tree Bagging, LR, and GBM	0.9164	0.0109
Code: Socializing with Positive Intent		
RF	0.8468	0.0326
SVM	0.8467	0.0321
Tree Bagging	0.8434	0.0363
LR	0.8360	0.0406
Naïve Bayes	0.8346	0.0380
GBM	0.8467	0.0276
Stacking of RF, SVM, Tree Bagging, LR, Naïve Bayes, and GBM	0.8836	0.0178
Code: Information Seeking		
RF	0.9484	0.0263
SVM	0.9465	0.0300
Tree Bagging	0.9345	0.0355
LR	0.9407	0.0308
Naïve Bayes	0.8935	0.0440
GBM	0.9495	0.0291
Stacking of RF, SVM, Tree Bag, LR, Naïve Bayes, and GBM	0.9750	0.0106
Code: Providing Resources		
RF	0.8116	0.0641
SVM	0.8025	0.0635
Tree Bagging	0.7730	0.0633
LR	0.7875	0.0725
Naïve Bayes	0.7638	0.0638
Stacking of RF, SVM, Tree Bagging, LR, and Naïve Bayes	0.8728	0.0192

## Appendix 2

**Table 6:** Lexical Features Predicting Codes of the ‘Learning in the Wild’ Schema.

Codes	LIWC Categories					
	Linguistic Processes	Other Grammar	Psychological Processes	Time Orientations	Personal Concerns	Punctuation
Explanation with Neutral Presentation	Analytic Authentic Words per sentence Words >6 letters Dictionary words Total function words Auxiliary verbs Prepositions	Common verbs Number	Power Drives Relativity	Past focus Space Time	Death Informal language Netspeak	All Punctuation Other punctuation Period Comma
Socializing with Positive Intent	Analytic Tone Words per sentence Words >6 letters Dictionary words 1st person singular	Common verbs	Positive emotion	Present focus	Work	Exclamation marks Other punctuation
Information Seeking	Analytic Words per sentence Words >6 letters Dictionary words Impersonal pronouns Auxiliary verbs	Common verbs Interrogatives	Social processes Tentative		Informal language Netspeak	Period Question marks Other punctuation
Providing Resources	Word count Analytic Clout Authentic Words per sentence Words >6 letters Dictionary words Total pronouns Articles Prepositions Auxiliary verbs	Common verbs Common adjectives Number	Affective processes Social processes Drives	Past focus Present focus Space Time	Informal language Netspeak	Period Comma Colon Question mark Other punctuation

## References

[CR1] Abaho, Michael, Daniel Gartner, Federico Ceruti, John Boulton (2018). Text Annotation using Textual Semantic Similarity and Term-Frequency (Twitter). In P. Bednar; U. Frank; and K. Kautz (eds): *ESCIS2018. Proceedings of the Twenty-Sixth European Conference on Information Systems, Portsmouth, UK, 23–28 June 2018*. AIS Electronic Library: Association for Information Systems, https://aisel.aisnet.org/ecis2018_rp/205.

[CR2] Abbar, Sofiane; Yelena Mejova; and Ingmar Weber (2015). You Tweet What You Eat: Studying Food Consumption Through Twitter. In B. Begole; J. Kim; K. Inkpen; and W. Woo (eds): *CHI’15. Proceedings of the 33rd Annual ACM Conference on Human Factors in Computing Systems, Seoul, Republic of Korea, 18 –23 April, 2015*. New York: ACM Press, pp. 3197–3206.

[CR3] Anderson T, Rourke L, Garrison DR, Archer W (2001). Assessing teaching presence in a computer conferencing context. Online Learning.

[CR4] Andrews R (2009). *Argumentation in Higher Education*.

[CR5] Andrews, Richard; and Caroline Haythornthwaite (2007). *The SAGE Handbook of E-learning Research*. London: SAGE.

[CR6] Baker M, Andriessen J, Lund K, van Amelsvoort M, Quignard M (2007). Rainbow: A framework for analysing computer-mediated pedagogical debates. International Journal of Computer-Supported Collaborative Learning.

[CR7] Bandura, Albert (1977). Self-Efficacy: Toward a Unifying Theory of Behavioral Change. *Psychological Review*, vol. 84, no 2, pp. 191–215. 10.1037//0033-295x.84.2.191 847061

[CR8] Birnbaum ML, Ernala SK, Rizvi AF, De Choudhury M, Kane JM (2017). A collaborative approach to identifying social media markers of schizophrenia by employing machine learning and clinical appraisals. Journal of Medical Internet Research.

[CR9] Bolognesi M, Pilgram R, van den Heerik R (2017). Reliability in content analysis: The case of semantic feature norms classification. Behavior Research Methods.

[CR10] Bruffee KA (1993). *Collaborative learning: Higher education, interdependence, and the authority of knowledge*.

[CR11] Buckingham Shum, Simon; and Rebecca Ferguson (2012). Social learning analytics. *Journal of Educational Technology & Society,* vol. 15, no. 3, pp. 3–26.

[CR12] Chen B, Bryer T (2012). Investigating instructional strategies for using social media in formal and informal learning. The International Review of Research in Open and Distributed Learning.

[CR13] Chugh R, Ruhi U (2018). Social media in higher education: A literature review of Facebook. Education and Information Technologies.

[CR14] Ferguson, Rebecca; Zhongyu Wei; Yulan He; and Simon Buckingham Shum (2013). An Evaluation of Learning Analytics to Identify Exploratory Dialogue in Online Discussions. In D. Suthers, K. Verbert, E. Duval, and X. Ochoa (eds): *LAK’13*. *Proceedings of the Third International Conference on Learning Analytics and Knowledge, Leuven, Belgium, 8–13 April, 2013.* New York: ACM Press, pp. 85–93.

[CR15] Flach, Peter; and Meelis Kull (2015). Precision-recall-gain curves: PR analysis done right. In C. Cortes; N.D. Lawrence; D.D. Lee; M. Sugiyama; and R. Garnett (eds): *NIPS 2015*. *Proceedings of the Neural Information Processing Systems 2015 Conference, Montreal, Canada, 7–12 December, 2015.* NIPS Proceedings: Neural Information Processing Systems Conference, pp. 838–846.

[CR16] Gilbert S (2016). Learning in a Twitter-based community of practice: an exploration of knowledge exchange as a motivation for participation in #hcsmca. Information, Communication & Society.

[CR17] Gilbert, Sarah (2018). *Motivations for participating in online initiatives: Exploring motivations across initiative types*. Ph.D. dissertation. University of British Columbia, Canada: Department of Library, Archival, and Information Studies.

[CR18] Goos, Merrilyn (2002) Understanding Metacognitive Failure. *The Journal of Mathematical Behavior,* vol. 21, no. 3, pp. 283-302.

[CR19] Greif, Irene. (1988). Overview. In Greif (ed.): *Computer-Supported Cooperative Work: A Book of Readings*. San Mateo, California: Morgan Kaufmann Publishers, pp. 5–12.

[CR20] Gruzd, Anatoliy; and Nadia Conroy (2020). Learning Analytics Dashboard for Teaching with Twitter. In T.X. Bui (eds): *HICSS 2020*. *Proceedings of the 53rd Hawaii International Conference on System Sciences, Maui, Hawaii, 7–10 January, 2020.* Scholar Space: University of Hawaii, pp. 2708- 2717. 10.24251/HICSS.2020.330.

[CR21] Gruzd A, Haythornthwaite C (2013). Enabling Community Through Social Media. Journal of Medical Internet Research.

[CR22] Gruzd A, Jacobson J, Wellman B, Mai P (2016). Understanding communities in an age of social media: the good, the bad, and the complicated, *Information*. Communication & Society.

[CR23] Gruzd, Anatoliy; Caroline Haythornthwaite; Drew Paulin; Sarah Gilbert; and Marc Esteve del Valle. (2018). Uses and gratifications factors for social media use in teaching: Instructors’ perspectives. *New Media and Society*, vol. 20, no. 2, pp. 475-494. 10.1177/1461444816662933

[CR24] Gunawardena CN, Lowe CA, Anderson T (1997). Analysis of a Global Online Debate and the Development of an Interaction Analysis Model for Examining Social Construction of Knowledge in Computer Conferencing. Journal of Educational Computing Research.

[CR25] Gunawardena CN, Hermans MB, Sanchez D, Richmond C, Bohley M, Tuttle R (2009). A theoretical framework for building online communities of practice with social networking tools. Educational Media International.

[CR26] Guzman, Emitza; Mohamed Ibrahim; and Martin Glinz (2017). A little bird told me: Mining tweets for requirements and software evolution. In A. Moreira; J. Araújo; J. Hayes; and B. Paech (eds): *2017 IEEE. Proceedings of the 25th International Requirements Engineering Conference, Lisbon, Portugal, 4–8 September, 2017.* IEEE Xplore Digital Library: IEEE, pp. 11–20.

[CR27] Hayes AF, Krippendorff K (2007). Answering the Call for a Standard Reliability Measure For Coding Data. Communication Methods and Measures.

[CR28] Haythornthwaite, Caroline; and Anatoliy Gruzd (2012). Exploring Patterns and Configurations in Networked Learning Texts. In R.H. Sprague, Jr. (eds): *HICSS 2012. Proceedings of the 45th Annual Hawaii International Conference on System Sciences, Maui, Hawaii, 4– 7 January, 2012*. IEEE Xplore Digital Library: IEEE, pp. 3358–67. 10.1109/HICSS.2012.268.

[CR29] Haythornthwaite, Caroline; Richard Andrews; Jude Fransman; and Eric M. Meyers (2016). The SAGE Handbook of E-learning Research. London: SAGE.

[CR30] Haythornthwaite C, Kumar P, Gruzd A, Gilbert S, del Valle ME, Paulin D (2018). Learning in the Wild: Coding for Learning and Practice on Reddit. Learning, Media and Technology.

[CR31] Hine C (2006). *New Infrastructures for Science Knowledge Production: Understanding E-Science*.

[CR32] Hoadley C, Kali Y, Kali Y, Baram-Tsabari A, Schejter AM (2019). Five waves of conceptualizing knowledge and learning for our future in a networked society. *Learning in a Networked Society: Spontaneous and Designed Technology Enhanced Learning Communities*.

[CR33] Johnson, Matthew; Dhiraj Murthy; Brett Roberstson; Roth Smith; and Keri Stephens (2020). DisasterNet: Evaluating the Performance of Transfer Learning to Classify Hurricane-Related Images Posted on Twitter. In T.X. Bui (eds): *HICSS 2020. Proceedings of the 53rd Hawaii International Conference on System Sciences, Maui, Hawaii, 7–10 January, 2020.* Scholar Space: University of Hawaii, pp. 576 – 583.

[CR34] Kaufhold, Marc-André, Markus Bayer, and Christian Reuter (2020). Rapid relevance classification of social media posts in disasters and emergencies: A system and evaluation featuring active, incremental and online learning. *Information Processing & Management*, vol. 57, no. 1, pp. 102132.

[CR35] Khazaei, Taraneh, Lu Xiao, and Robert Mercer (2017). Writing to persuade: Analysis and detection of persuasive discourse. In W. Sterzer (ed): *iConference 2017*. *Proceedings of the 2017 iConference, Wuhan, China, 22–25 March, 2017*. IDEALS: iSchools, pp. 203-215. http://hdl.handle.net/2142/96673

[CR36] Koschmann T (1996). *CSCL: Theory and Practice of an Emerging Paradigm*.

[CR37] Koschmann T, Hall R, Miyake N (2002). *CSCL 2: Carrying Forward the Conversation*.

[CR38] Krippendorff K (2004). Reliability in content analysis: Some common misconceptions and recommendations. Human Communication Research.

[CR39] Kumar, Priya; and Anatoliy Gruzd (2019). Social Media for Informal Learning: A Case of #Twitterstorians. In T.X. Bui (eds): *HICSS 2019. Proceedings of the 52nd Annual Hawaii International Conference on System Sciences, Maui, Hawaii, 8–11 January, 2019.* Scholar Space: University of Hawaii, pp. 2527-2535. 10.24251/HICSS.2019.304.

[CR40] Kumar, Priya; Anatoliy Gruzd; Caroline Haythornthwaite; Sarah Gilbert; Marc Esteve del Valle; and Drew Paulin (2018). Learning in the Wild: Coding Reddit for Learning and Practice. In T.X. Bui (eds): *HICSS 2018. Proceedings of the 51st Annual Hawaii International Conference on System Sciences (HICSS), Big Island, Hawaii, 2–6 January, 2018.* Scholar Space: University of Hawaii, pp 1933-1942. 10.24251/HICSS.2018.244.

[CR41] Laat D, Maarten, Lally V (2003). Complexity, theory and praxis: Researching collaborative learning and tutoring processes in a networked learning community. Instructional Science.

[CR42] Lave, Jean; and Etienne Wenger (1991). *Situated Learning: Legitimate Peripheral Participation*. Cambridge University Press.

[CR43] Lewis, Bex; and David Rush. (2013). Experience of developing Twitter-based communities of practice in higher education. *Research in Learning Technology*, vol. 21, no. 1, pp. 18598 - 10.3402/rlt.v21i0.18598

[CR44] Loudon, Mellisa. (2014). ‘Research in the wild’ in online communities: Reddit’s resistance to SOPA. *First Monday*, vol. 19, no. 2, doi: 10.5210/fm.v19i2.4365.

[CR45] Mercer N (2004). Sociocultural discourse analysis: analysing classroom talk as a social mode of thinking. Journal of Applied Linguistics.

[CR46] Nardi, Bonnie A.; Steve Whittaker; and Heinrich Schwarz (2002). NetWORKers and their Activity in Intensional Networks, *Computer Supported Cooperative Work (CSCW)*, vol. 11, no. 1-2, pp. 205-242.

[CR47] Nayak A, Natarajan S (2016). Comparative Study of Naïve Bayes, Support Vector Machine and Random Forest Classifiers in Sentiment Analysis of Twitter Feeds. International Journal of Advance Studies in Computer Science and Engineering (IJASCSE).

[CR48] Orlikowski WJ (2002). Knowing in practice: Enacting a collective capability in distributed organizing. Organization Science.

[CR49] Paulin D, Gilbert S, Haythornthwaite C, Andrews R, Fransman J, Meyers EM (2016). Social media and learning. *The SAGE Handbook of E-learning Research*.

[CR50] Paulin D, Haythornthwaite C (2016). Crowdsourcing the curriculum: Redefining e-learning practices through peer-generated approaches. The Information Society.

[CR51] Pena-Shaff JB, Nicholls C (2004). Analyzing student interactions and meaning construction in computer bulletin board discussions. Computers & Education.

[CR52] Preece J (2000). *Online communities: designing usability and supporting sociability*.

[CR53] Reed, Peter. (2013). Hashtags and retweets: using Twitter to aid community, communication and casual (informal) learning, *Research in Learning Technology*, vol. 21, no. 1, pp. 19602, 10.3402/rlt.v21i0.19692.

[CR54] Renninger KA, Shumar W (2002). *Building Virtual Communities: Learning and Change in Cyberspace*.

[CR55] Resnik, Philip, William Armstrong, Leonardo Claudino, Thang Nguyen, Viet-An Nguyen, and Jordan Boyd-Graber. (2015). Beyond LDA: Exploring supervised topic modeling for depression-related language in Twitter. In M. Mitchell; G. Coppersmith; and K. Hollingshead (eds): *NAACL HLT 2015*. *Proceedings of the 2nd Workshop on Computational Linguistics and Clinical Psychology: From Linguistic Signal to Clinical Reality, Denver, Colorado, 5 June, 2015*. New York: Association for Computational Linguistics, pp. 99–107.

[CR56] Schmidt K, Bannon L (2013). Constructing CSCW: The first quarter century. Computer Supported Cooperative Work (CSCW).

[CR57] Sengupta, Subhasree; and Caroline Haythornthwaite. (2019). *Enhancing quality of content on Stack Overflow - a preliminary analysis of SO comments* [Poster Presentation]. the 10th International Conference on Social Media and Society, Toronto, ON.

[CR58] Shane-Simpson C, Manago A, Gaggi N, Gillespie-Lynch K (2018). Why do college students prefer Facebook, Twitter, or Instagram? Site affordances, tensions between privacy and self-expression, and implications for social capital. Computers in Human Behavior.

[CR59] Siemens, George (2005). Connectivism: A learning theory for the digital age. *International Journal of Instructional Technology and Distance Learning,* vol. 2, no. 1, http://www.itdl.org/journal/jan_05/article01.htm

[CR60] Sokolova M, Lapalme G (2009). A systematic analysis of performance measures for classification tasks. Information Processing & Management.

[CR61] Stahl G, Koschmann TD, Suthers DD, Sawyer RK (2006). Computer-supported collaborative learning: An historical perspective. *Cambridge handbook of the learning sciences*.

[CR62] Stanik, Christoph; Marlo Haering; and Walid Maalej (2019). Classifying Multilingual User Feedback using Traditional Machine Learning and Deep Learning. In A. Perini; and D. Damian (eds): *2019 IEEE. Proceedings of the 27th International Requirements Engineering Conference Workshops (REW), Jeju Island, South Korea, 23–27 September, 2019.* IEEE Xplore Digital Library: IEEE, pp. 220–226.

[CR63] Stowe K, Anderson J, Palmer M, Palen L, Anderson KM, Ku L, Li C (2018). Improving classification of twitter behavior during hurricane events. *SocialNLP*. *Proceedings of the Sixth International Workshop on Natural Language Processing for Social Media, Melbourne, Australia, 20 July, 2018*.

[CR64] Sylwester K, Purver M (2015). Twitter language use reflects psychological differences between democrats and republicans. PloS one.

[CR65] Tausczik YR, Pennebaker JW (2010). The psychological meaning of words: LIWC and computerized text analysis methods. Journal of language and social psychology.

[CR66] Valle D, Esteve M, Gruzd A, Kumar P, Gilbert S, Dohn NB, Jandrić P, Ryberg T, Laat M (2020). Learning in the Wild: Understanding Networked Ties in Reddit. *Mobility, Data and Learner Agency in Networked Learning*.

[CR67] Veldhuis-Diermanse, Anna Elske (2002). *CSCLearning?: participation, learning activities and knowledge construction in computer-supported collaborative learning in higher education*. Ph.D. dissertation. Wageningen University, Netherlands: Department of Education and Learning Sciences.

[CR68] Veletsianos G (2012). Higher education scholars’ participation and practices on Twitter. Journal of Computer Assisted Learning.

[CR69] Weinberger A, Fischer F (2006). A framework to analyze argumentative knowledge construction in computer-supported collaborative learning. Computers & Education.

[CR70] Wenger E (1998). *Communities of practice: Learning, meaning, and identity*.

[CR71] Williams, Grant; and Mahmoud, Anas (2017). Mining Twitter feeds for software user requirements. In A. Moreira; J. Araújo; J. Hayes; and B. Paech (eds): *2017 IEEE. Proceedings of the 25th International Requirements Engineering Conference, Lisbon, Portugal, 4–8 September, 2017.* IEEE Xplore Digital Library: IEEE, pp. 1–10.

[CR72] Wise, Alyssa Friend; Simone Nicole Hausknecht; and Yuting Zhao (2014). Attending to others' posts in asynchronous discussions: Learners' online "listening" and its relationship to speaking. International Journal of Computer-Supported Collaborative Learning, vol. 9, no. 2, pp. 185-209.

[CR73] Wobbrock JO, Kientz JA (2016). Research contributions in human-computer interaction. Interactions.

[CR74] Zhao Y, Guo Y, He X, Wu Y, Yang X, Prosperi M (2019). Assessing Mental Health Signals Among Sexual and Gender Minorities using Twitter Data. Health Informatics Journal.

